# Right pulmonary artery originating from the ascending aorta with tetralogy of fallot and pulmonary atresia

**DOI:** 10.1002/ccr3.9232

**Published:** 2024-08-12

**Authors:** Filippos‐Paschalis Rorris, Meletios Kanakis, George Samanidis, Alexandros Tsoutsinos, Achilleas Lioulias, Dimitrios Bobos

**Affiliations:** ^1^ Department of Paediatric and Adult Congenital Heart Surgery Onassis Cardiac Center Athens Greece; ^2^ 1st Department of Adult Cardiac Surgery Onassis Cardiac Surgery Center Athens Greece; ^3^ Department of Paediatric Cardiology and Adult Congenital Heart Disease Onassis Cardiac Surgery Centre Athens Greece

**Keywords:** congenital heart defect, Hemitruncus, pulmonary artery from ascending aorta, pulmonary atresia

## Abstract

**Key Clinical Message:**

Anomalous origin of right pulmonary artery from the ascending aorta is a rare congenital heart malformation that results in early infant mortality. These patients are at risk for the early development of significant pulmonary hypertension. The surgical management during the early period of life is imperative.

**Abstract:**

Anomalous pulmonary artery originating from the ascending aorta (often called hemitruncus) is a rare congenital cardiac defect requiring immediate management in the neonatal period. We report a case of a rare variant of anomalous right pulmonary artery originating from the ascending aorta in combination with pulmonary atresia and tetralogy of Fallot. The above‐mentioned combination makes the surgical management of such cardiac defect exceedingly demanding.

A neonate was transferred immediately after birth in our Pediatric and Congenital Cardiac Service for accurate diagnosis and subsequent management of a complex cardiac defect. The patient was in heart failure. Preoperative echocardiogram as well as computed tomography (CT) scan with 3D reconstruction (Figure 1A,B red arrow) revealed a right pulmonary artery originating from the ascending aorta, pulmonary atresia with hypoplastic main pulmonary trunk, an atrial septal defect (ASD) as well as a ventricular septal defect (VSD), and an overring aorta. The diagnosis was confirmed intraoperatively (Figure 1C,D black arrow).

**FIGURE 1:**
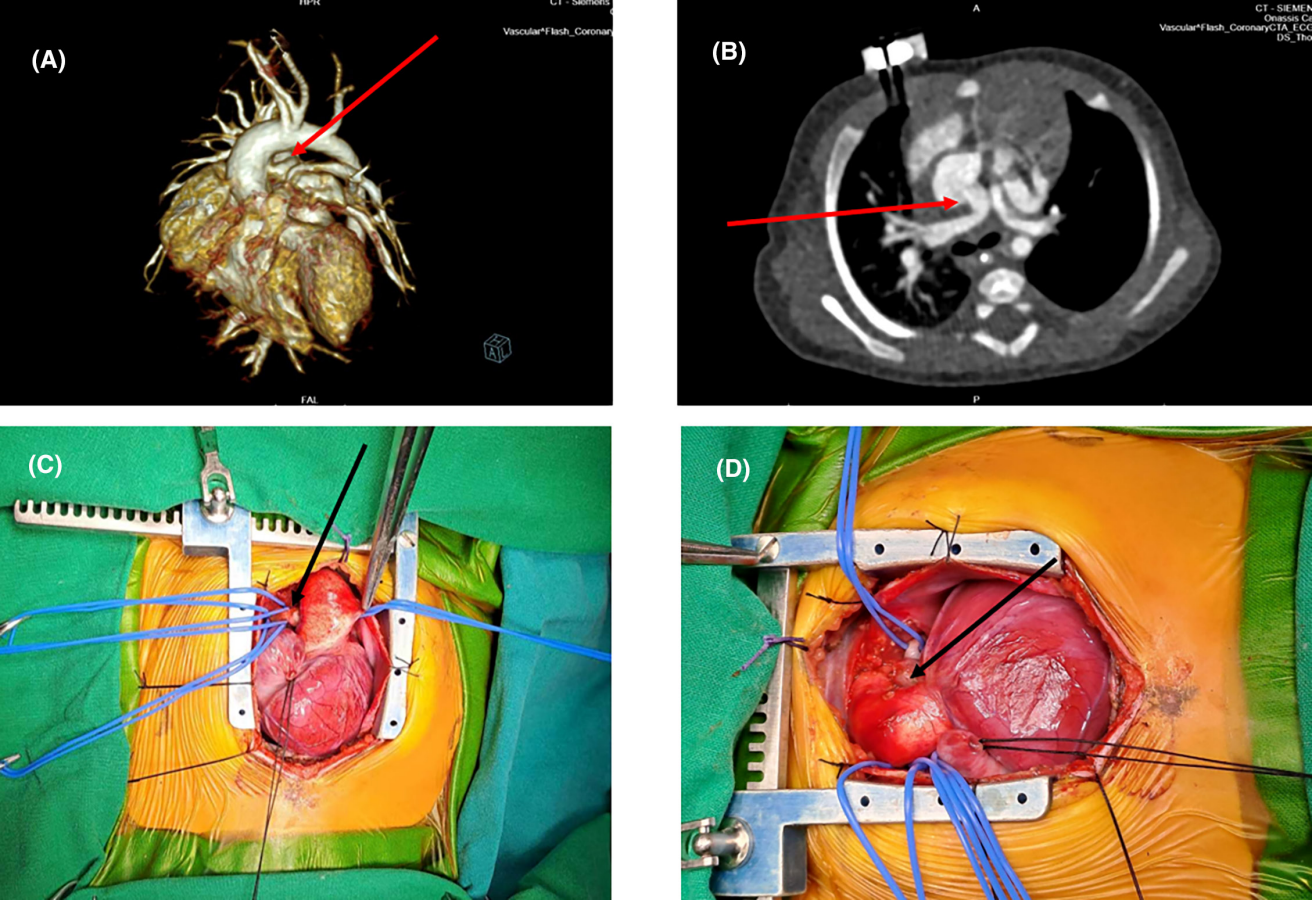
Computed tomography scan with 3D reconstruction and intraoperative images; showing the anomalous right pulmonary artery originating from the ascending aorta (A, B red arrow and C, black arrow). Inlet image (D) showing the atretic pulmonary artery (black arrow).

After completing the pre‐operative assessment, the neonate was scheduled for a first stage surgical management. Under cardiopulmonary bypass (CPB) and mild hypothermia, the anomalous right pulmonary artery was transected from the ascending aorta and anastomosed to the right lateral side of the main pulmonary trunk. The obstruction was observed at the pulmonary valvar level and, as such, the pulmonary valve leaflets were divided to relieve the obstruction and create subsequent forward flow to the pulmonary vessels. The main pulmonary trunk was augmented using bovine pericardium. During CPB weaning, the blood oxygen saturations were not satisfying and therefore we proceeded with creating an aortopulmonary anastomosis (modified Blalock‐Taussig shunt / mBTS). CBP weaning was successful, and the neonate was transferred to the cardiac Intensive Care Unit (ICU). The neonate's postoperative course was uneventful and was discharged home and is planned for a final surgical management which includes mBTS take down, closure of VSD and creation of a large right ventricle to pulmonary artery patch.

Anomalous origin of right pulmonary artery from the ascending aorta is a rare congenital heart malformation that results in early infant mortality affecting the right pulmonary artery more than the left (right or left “hemitruncus”).^1^ These patients are at risk for the early development of significant pulmonary hypertension. The surgical management during the early period of life is imperative.^2^ Accurate diagnosis is of outmost importance for the subsequent precise surgical management.

## AUTHOR CONTRIBUTIONS

**Filippos‐Paschalis Rorris:** Investigation; writing – original draft. **Meletios Kanakis:** Investigation; methodology; supervision; visualization; writing – original draft. **George Samanidis:** Visualization; writing – original draft. **Alexandros Tsoutsinos:** Investigation. **Achilleas Lioulias:** Investigation. **Dimitrios Bobos:** Investigation.

## FUNDING INFORMATION

N/A.

## CONSENT

Written informed consent was obtained from the parent of children to publish this report in accordance with the journal's patient consent policy.

## Data Availability

The data that support the findings of this study are available on request from the corresponding author.
